# Functional Profiling of Unfamiliar Microbial Communities Using a Validated *De Novo* Assembly Metatranscriptome Pipeline

**DOI:** 10.1371/journal.pone.0146423

**Published:** 2016-01-12

**Authors:** Mark Davids, Floor Hugenholtz, Vitor Martins dos Santos, Hauke Smidt, Michiel Kleerebezem, Peter J. Schaap

**Affiliations:** 1 Laboratory of Systems and Synthetic Biology, Wageningen University, Dreijenplein 10, Wageningen, The Netherlands; 2 Laboratory of Microbiology, Wageningen University, Dreijenplein 10, Wageningen, The Netherlands; 3 Host-Microbe Interactomics Group, Wageningen University, Wageningen, The Netherlands; 4 Netherlands Consortium for Systems Biology, TI Food and Nutrition, Wageningen, The Netherlands; Georgia Institute of Technology, UNITED STATES

## Abstract

**Background:**

Metatranscriptomic landscapes can provide insights in functional relationships within natural microbial communities. Analysis of complex metatranscriptome datasets of these communities poses a considerable bioinformatic challenge since they are non-restricted with a varying number of participating strains and species. For RNA-Seq data a standard approach is to align the generated reads to a set of closely related reference genomes. This only works well for microbial communities for which a near complete catalogue of reference genomes is available at a small evolutionary distance. In this study, we focus on the design of a validated *de novo* metatranscriptome assembly pipeline for single-end Illumina RNA-Seq data to obtain functional and taxonomic profiles of murine microbial communities.

**Results:**

The here developed *de novo* assembly metatranscriptome pipeline combined rRNA removal, IDBA-UD assembler, functional annotation and taxonomic classification. Different assemblers were tested and validated using RNA-Seq data from an *in silico* generated mock community and *in vivo* RNA-Seq data from a restricted microbial community taken from a mouse model colonized with Altered Schaedler Flora (ASF). Precision and recall of resulting gene expression, functional and taxonomic profiles were compared to those obtained with a standard alignment method. The validated pipeline was subsequently used to generate expression profiles from non-restricted cecal communities of four C57BL/6J mice fed on a high-fat high-protein diet spiked with an RNA-Seq data set from a well-characterized human sample. The spike in control was used to estimate precision and recall at assembly, functional and taxonomic level of non-restricted communities.

**Conclusions:**

A generic *de novo* assembly pipeline for metatranscriptome data analysis was designed for microbial ecosystems, which can be applied for microbial metatranscriptome analysis in any chosen niche.

## Background

High throughput metagenomics have revolutionized our knowledge of microbial communities such as those that populate the human gastrointestinal (GI) tract. Complementing 16S ribosomal RNA gene-based compositional analyses, metagenome sequencing of these communities provided a broad description of the genetic content and relative abundance of individual members [1–6]. The human enterotypes, for instance, have been defined using comparative metagenomic analysis of the human gut microbiomes of 39 individuals [5]. Metagenomics, however, does not provide insights in the functional interactions within a complex microbial ecosystem and how these interactions may change in response to an ever-changing environment, including diet. RNA transcript profiling can fill this gap and serve as a proxy for ecosystem responses to environmental cues. Recent advances in massive parallel sequencing of mRNA-derived cDNA sequences (RNA-Seq) has led to an exponential increase of such transcriptome profiling studies. While most RNA-Seq based expression studies focus on a single species, in a number of cases RNA-Seq has been used to profile complex natural microbial communities in marine, soil and human and other mammalian GI tract environments [7–15]. Analysis of these large complex datasets poses a considerable bioinformatic challenge since natural microbial communities are usually non-restricted with a varying number of participating strains and species. A standard approach is to align the generated RNA-Seq reads to a set of closely related reference genomes or well-annotated metagenomes [10,13,14]. This approach works well for the well-studied microbial communities that have a nearly complete catalogue of reference genomes at a small evolutionary distance available [10]. However, at a larger evolutionary distance, the extensive sequence diversity at nucleotide level between the sample and the reference database significantly reduces the mapping efficiency of the alignment method and increases the probability of spurious assignments.

To overcome these problems a *de novo* assembly method can be used. *De novo* assembly of RNA-Seq reads into contigs increases the information content and therefore grants a more reliable annotation of the expressed genetic content of an unknown microbial community [16,17]. Subsequently the newly assembled contigs can be directly used as target sequences in an mRNA-read mapping approach to obtain gene expression data. Currently a number of de Bruijn graph based assemblers have been developed for *de novo* assembly of Illumina sequencing data [18,19]. Most of them have been designed to work with genomic data from a single species and assume that reads are uniformly sampled along a length of a single genome. As such they cannot efficiently deal with the existence of many co-linear genomic regions in the genomes of strains and species encountered in a non-restricted natural microbial community [16,17]. Sequencing errors, exacerbated by authentic microdiversity caused by the coexistence of syntenic strains of the same species in a community and strong sequence conservation of genes common to many species in the community thus can lead to assemblies with a relatively high rate of small contigs and to ambiguous chimeric contigs. Due to the limited size and strong variations in read coverage, statistical analysis methods to assess the correctness of *de novo* metagenome assemblies’ will not reliably work for RNA-Seq derived contigs. Consequently additional verification strategies, such as PCR, are necessary as confirmation of the genetic context predicted by assembled contigs.

The microbiome of the GI tract of healthy human individuals fulfils a variety of beneficial functions for human health [20]. Numerous studies have linked an altered gut microbiome to disorders in energy and metabolic homeostasis including obesity and diabetes, as well as immune aberrations and excessive inflammation diseases [21–23]. For a systematic study of the influence of diet, environmental factors and host genotype on the microbial diversity in the GI tract, animal models provide an indispensable tool. To this end the mouse model has emerged as one of the preferred model systems. Mouse intestinal microbial communities have been mapped using 16S rRNA gene-based community profiling, and many microbial mouse intestinal commensals have been identified and categorized. Although the phylogenetic makeup of the GI tract microbial communities in human and mouse appear to be similar at phylum level, zooming in at genus and species resolution reveals considerable differences in bacterial composition [24,25]. The large evolutionary distance of the microorganisms, combined with a strong bias towards human microbiome sequences in the current GI gene catalogs results, as we will show here, in low-resolution outcomes of the analysis of mouse metatranscriptome data with standard alignment methods [13]. Moreover previously single- and paired-end sequencing methods indicated that paired-end sequencing does not gain in performance [10]. This led us to design, validate and implement a *de novo* assembly pipeline for single-end Illumina sequencing using existing assemblers. The method provides better gene assignment results then alignment methods, and we evaluated sensitivity, reliability and validity of the method for the function analysis of complex metatranscriptome data. The generic *de novo* assembly method developed was validated by PCR and using metatranscriptome datasets of community-restricted samples and a spike-in of a human control sample, and enabled a reliable functional profiling and taxonomic binning of samples obtained from the mouse GI tract.

## Results and Discussion

### General Workflow, Samples and Data Filtering

**Fig 1:**
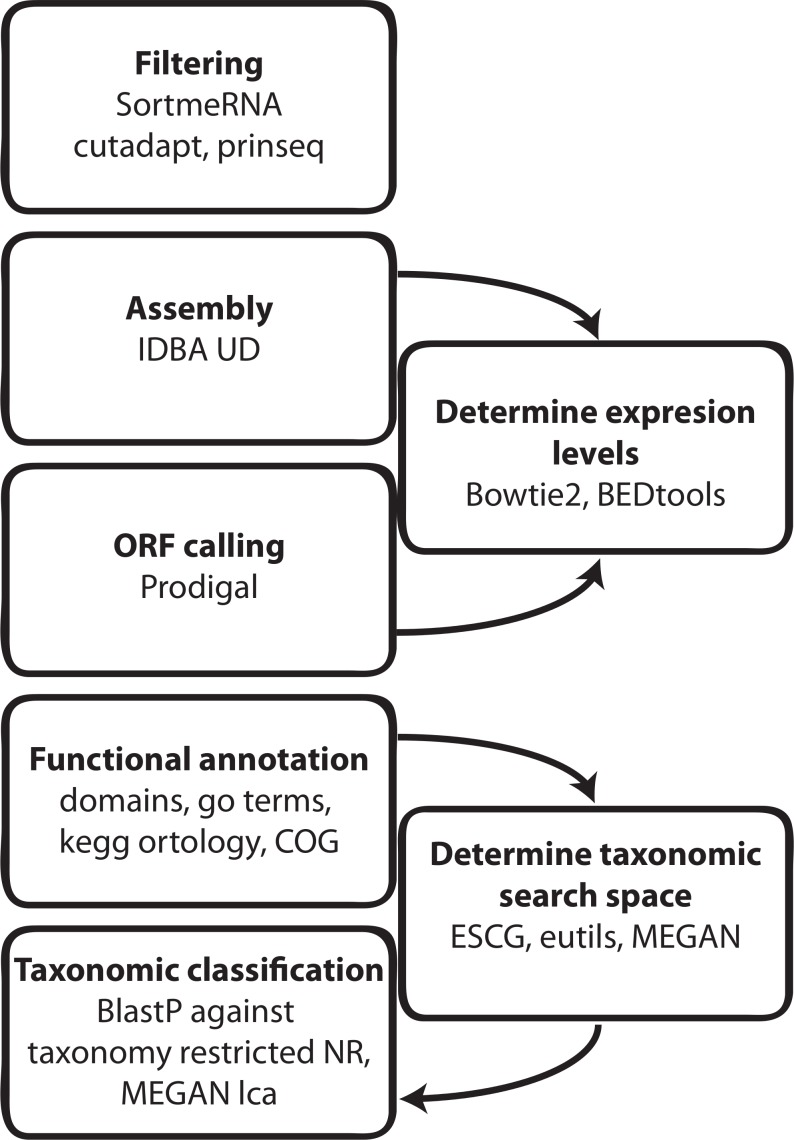
Metatranscriptome analysis workflow. Details of the programs used are described in the methods section.

A generalized metatranscriptome assembly and analysis pipeline was designed (Fig 1). Briefly the workflow consisted of filtering RNA-Seq reads for low quality and non-informative reads such as reads derived from ribosomal RNA (rRNA) followed by assembly of the remaining putative ‘mRNA’ reads into contigs, ORF calling and gene-function annotation, taxonomic classification and estimation of gene expression levels by using read-frequency analyses. Since no dedicated metatranscriptome assemblers have been developed so far, performances of several assemblers were evaluated. We have found that the output of the IDBA-UD assembler [26] was most appropriate for single end metatranscriptome data. Filtering details and assembler performances can be found in S1 File.

In order to validate the proposed pipeline, two single-end RNA-Seq metatranscriptome datasets were used. The first RNA-Seq dataset, obtained from an *in silico* generated mock community composed of eight strains, was used for a primary evaluation of the entire workflow. The second RNA-Seq dataset used to further validate the workflow resulted from an *in vivo* study of a restricted intestinal microbial community of limited complexity obtained by the colonization of inbred non-obese diabetic (NOD) mice with Altered Schaedler Flora (ASF) [13]. The datasets were used to estimate precision and recall of the metatranscriptome assembly procedure for functional and taxonomic level assignments respectively. Finally, the pipeline was used to generate functional and taxonomic community profiles of RNA-Seq datasets from natural microbial cecal communities of four C57BL/6J mice that were fed a high-fat high protein diet [27]. To estimate precision and recall of *de novo* assemblies obtained from this complex non-restricted mouse community, RNA-Seq data from a well-characterized sample from the human small intestine [10] was used as spike-in control. Technical replicates were available for one of the mouse samples and for the human small intestine sample and included to establish the technical reproducibility of the procedure. Furthermore, PCR followed by Sanger sequencing of the amplified product confirmed the correctness of the sequence of twelve randomly selected mouse derived transcript assemblies.

### Metatranscriptome assembly of an *in silico* mock community

An *in silico* mock metatranscriptome was built by merging RNA-Seq data of eight single species transcriptome profiling experiments downloaded from public repositories (Table 1). For each of the selected species a high quality reference genome was available, and RNA-Seq datasets selected were obtained with the Illumina HTS platform using 68 to 107 sequencing cycles. From paired-end datasets an arbitrarily selected single-end dataset was selected. To capture some of the complexity of a true unrestricted community three closely related species from the genus *Streptococcus* where chosen and mixed with five species at a larger evolutionary distance. Furthermore, in this mock community the number of mRNA reads of each of the eight added members was varied mimicking a high variation in species abundance and activity (Table 1).

**Table 1:** Composition of the mock metatranscriptome RNA-Seq dataset and assembly results.

Species	# filtered reads	Relative read abundance (%)	Assembled reads**	Sample ID
*Streptococcus agalactiae*	3,224,516	35.0	(98.0%)	SRR922307[28]
*Clostridium beijerinckii*	1,586,292	17.2	(95.2%)	SRR988002[29]
*Pediococcus claussenii*	1,371,187	14.9	(95.8%)	SRR647762[30]
*Streptococcus pneumoniae*	1,235,598	13.4	(97.2%)	SRR1009263
*Enterococcus faecium*	667,246	7.2	(89.8%)	SRR922448[31]
*Lactobacillus casei*	500,000	5.4	(90.0%)	SRR616266
*Streptococcus thermophilus*	396,951	4.3	(87.6%)	SRR390316
*Clostridium difficile*	239,138	2.6	(62.7%)	ERR406251*
**Overall**	9,220,928		(94.7%)	

* Pre submission data taken from http://www.sanger.ac.uk/datasharing/

** Assembly results presented were obtained by using the IDBA-UD assembler

Since the mRNA reads in the mock community dataset originate from a specific set of known genomes, the output of the *de novo* assembly workflow can be directly compared with results obtained from a standard alignment procedure. In total 8943 contigs were obtained with the IDBA-UD assembler and their precision at sequence level was assessed by aligning these sequences to the reference genomes. For 86% of these contigs an unambigous high quality full length sequence alignment without insertions or deletions to a reference genome was obtained (S1 File). The remaining contigs showed a varying degree of nucleotide mismatches at the 5’- and 3’-end of the contig sequences. Since most of these discrepancies were small, the majority of these remaining contigs could still be used for further functional profiling (see below). Only 2% of the assemblies were recognized as a cross-species assembly. Manual inspection showed that these chimeras aligned to sequences that had high levels of conservation among the most closely related species (S1 File) suggesting that sequence micro-diversity does not have a major impact on the assembly performance, and that a taxonomic classification of metatranscriptome assemblies at genus level and above should be possible. mRNA reads that were not used in the assemblies (Table 1) were analysed by a direct alignment with the corresponding reference genomes. In many cases these reads mapped to genes with a small open reading frame (< 100 amino acids) and transcripts of low abundance (data not shown). On basis of our analyses, we estimated that for this mock community a threefold coverage of a gene is required for at least a partial transcript assembly (S1 File).

**Fig 2:**
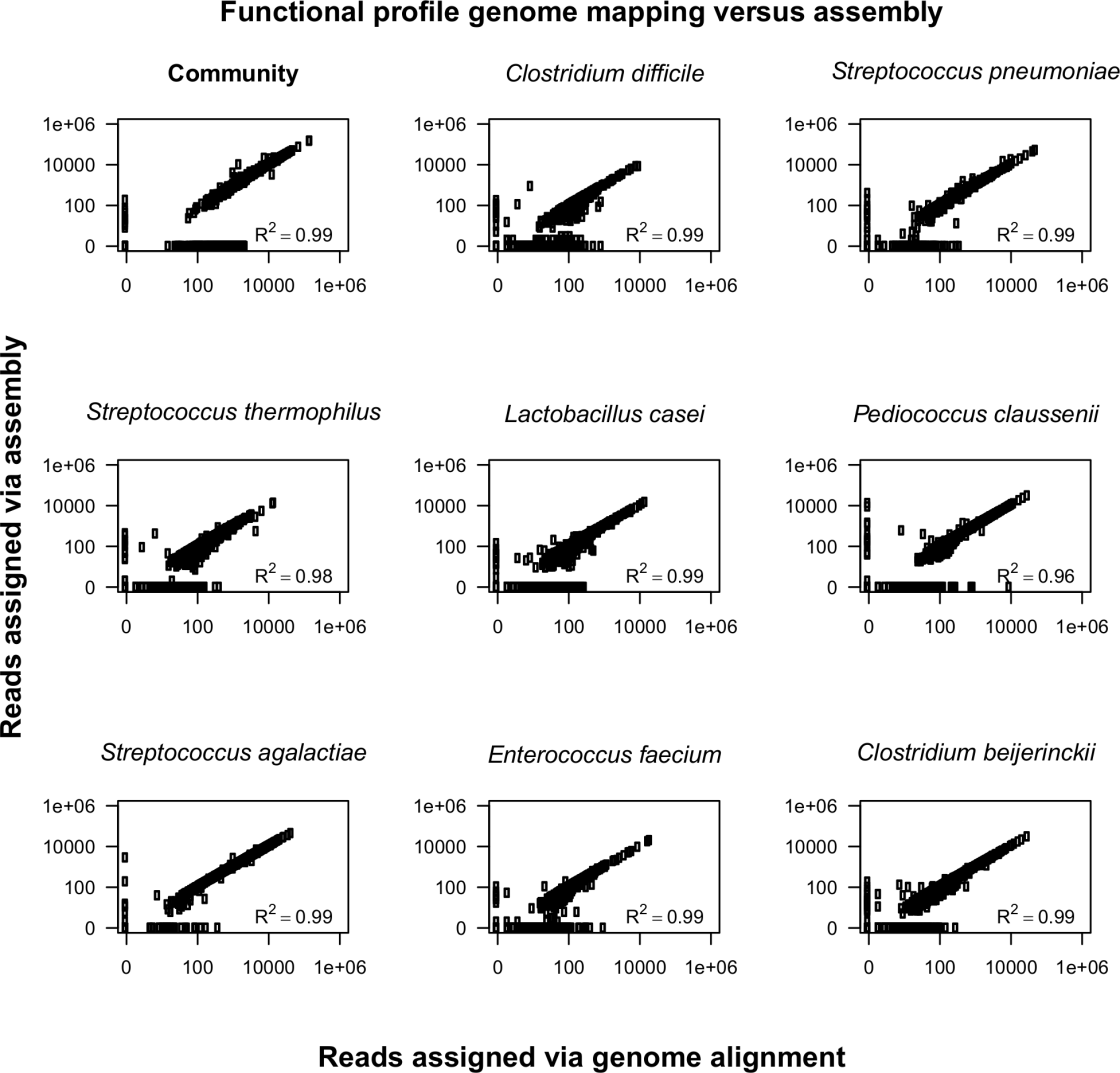
Comparison of functional profiles of an eight species mock community metatranscriptome. Reads assigned via direct genome alignment method (x-axis) and *de novo* assembly with IDBA-UD (y-axis). Each dot represents a specific KEGG orthologous function.

To determine the accuracy of the functional profiles obtained by *de novo* assembly, they were compared to profiles obtained by direct genome mapping. Results showed a high congruency (Pearson correlation > 0.99) for the mock community as a whole as well as for each of the individual members (Fig 2). We compared the KEGG orthology assignment for each read to determine the precision and recall. For functional prediction of assembly assigned reads we calculated a precision score of 0.97 with a recall of 0.94.

### Metatranscriptome assembly of Altered Schaedler Flora from the intestines of a NOD mouse model

For further validation of the pipeline a published RNA-Seq dataset obtained from a defined *in vivo* mouse intestinal community was used [13]. In this study RNA-Seq data was obtained from four inbred non-obese diabetic (NOD) germ-free mice colonized with a defined mixture of eight commensal bacteria (Altered Schaedler Flora; ASF). Twelve cecum and colon samples were prepared using multiple RNA-extraction protocols and sequenced using the Illumina HTS platform. When the RNA-Seq set was published [13] the complete set of ASF genomes had not been sequenced yet, and to bridge the evolutionary distance to known species a peptide-based alignment procedure was used for functional profiling. Using this procedure the authors were able to link 16% of the sequence reads to a known bacterial gene. Moreover using the Trinity assembler the annotated fractions could be increased to 50.3% [16]. Recently, the draft genome sequences of all eight bacteria in the ASF community have been determined [32], providing the opportunity to validate the metatranscriptome assembly pipeline with a restricted *in vivo* RNA-Seq dataset from a mouse intestinal community.

**Fig 3:**
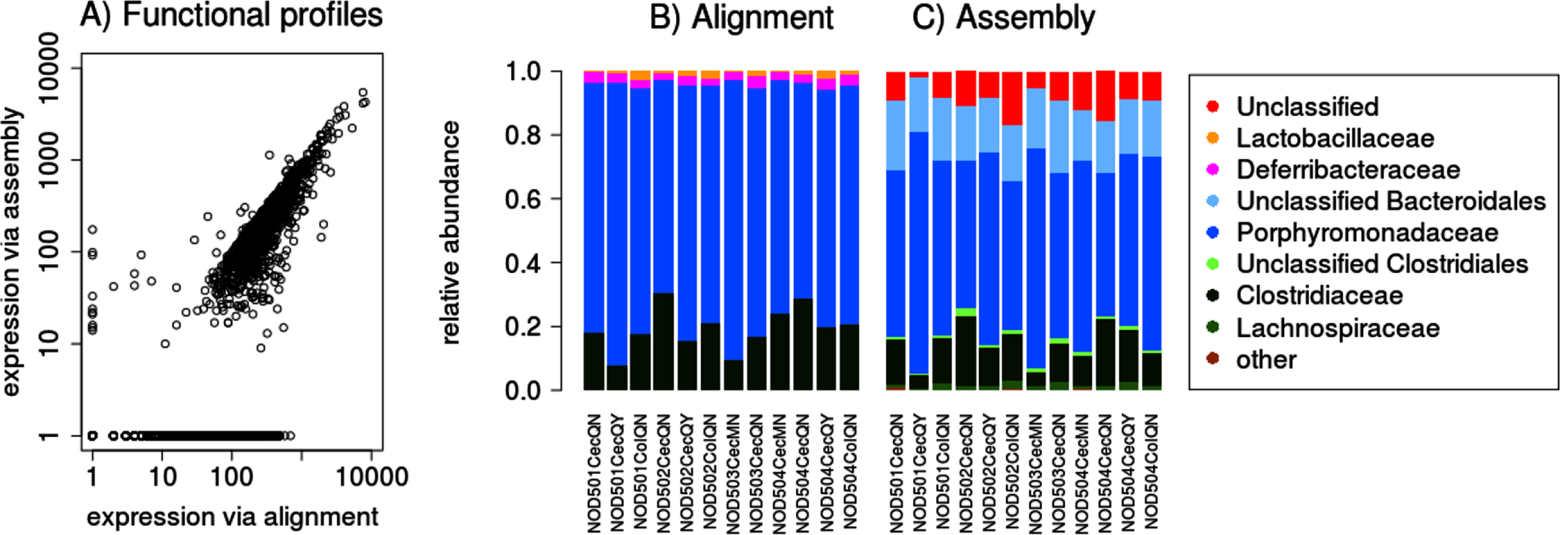
Comparison of functional and taxonomic profiles of the Altered Schaedler Flora from the intestine of a NOD mouse model [13]. A) Alignment vs assembly functional profiling; x-axis, direct genome alignment; y-axis, *de novo* assembly. Taxonomic profiles of mRNA reads obtained by direct genome mapping (B) and by using the *de novo* assembly method (C). Sample labels were taken from Xiong et al.

ASF reads were taken from the SRA repository (SRP012007) [13], and approximately 5,4 million reads of high quality passed the filtering stage of the pipeline. 1,7 million of these reads could be directly mapped on the recently sequenced draft ASF genomes while 1,5 million reads mapped to the host genome. The origin of the remaining 2,2 million reads could not be established. A blastx search of those unmapped reads against the NR database did not return any significant results. All 5,4 million reads were used for assembly with IDBA-UD to yield 7160 assembled transcripts. The majority (6638) of these contigs could be accurately aligned with one of the eight ASF draft genomes and 256 contigs were derived from the host leaving 266 of the contigs (4%) unaccounted for. The ASF-mapped contigs captured 8688 ORFs that were functionally annotated and compared with a direct mRNA read alignment to the annotated ASF draft genomes following the procedures described above. The result showed a strong correlation (pearson correlation > 0.9) between the expression values obtained from the assembly and the direct-alignment method (Fig 3).

Even though there were many contaminating reads in these samples that could interfere with the assembly process only a few false positive reads assignments were detected. The precision and recall for functional annotation was 0.97 and 0.58 respectively. The precision was therefore comparable to what was observed for the mock community, whereas the recall is much lower, which is probably due to a much lower sequencing depth of ASF samples (S1 File).

Additionally we explored the performance of a taxonomic classification of *de novo* assembled sequences. For a taxonomic classification a two-step procedure was developed. First, from the set of *de novo* assembled sequences from the ASF microbiome a subset of 103 proteins derived from essential single copy genes (ESCG) [33] was identified. This set of marker proteins was used to determine the boundaries of the taxonomic search space by aligning them with the entire NR protein database and further classification using the MEGAN processing pipeline [34] (see methods section for details), In order to mimic the lack of good reference genomes ASF derived protein sequences were excluded. Sixty-seven of 103 marker proteins were classified as proteins belonging to the order of Bacteroidales while 33 belonged to the Clostridiales. Only three marker proteins could not be classified at the order level, and therefore this taxonomic rank was used to restrict the reference database. With the taxonomic boundaries set to the Clostridiales and Bacteroidales, next non-ESCG proteins were classified by aligning them with all Clostridiales and Bacteroidales proteins present in the NR database, again excluding ASF proteins, and classification with MEGAN (Fig 3). The use of ESCG derived marker proteins to restrict the search space drastically reduced the computational time to classify the full set of proteins. However, low abundant species of different orders may be missed. In this case the presence of members of the Lactobacillales and Defferibacterales, which made up six percent of the mRNA reads in total, was not detected with this method.

Xiong *et al*. [13]used a blastx based alignment procedure to taxonomically assign mRNA reads of the ASF to known bacterial genes. Due to the small read length this method can give rise to many ambiguous assignments. Celaj *et al* showed that the assignment could be substantially improved by using a *de novo* assembly approach [16]. Moreover with large sets of mRNA reads assigned to a taxonomic rank via genome and assembly mapping we can estimate the precision and recall for the assignments via assembly. We assumed that the taxonomic association of a read obtained by a direct genome alignment to an ASF gene summarized in Fig 3 is true. If via the *de novo* assembly method a different taxonomic association was obtained it was considered to be a false positive association and in case such a read was not incorporated by the *de novo* assembly method or ended up in a taxonomically unclassified contig, it was considered to be a false negative association. For the correctness of a taxonomic classification of ASF proteins via the *de novo* assembly method we estimated a precision of 0.95 for taxonomic ranks down to genus level. The recall score for phylum to order level was 0.66 but was reduced to 0.38 for lower taxonomic ranks (genus & family) (S1 File). Relative abundance of community members was in very good agreement with those obtained by direct genome mapping (Fig 3).

### Metatranscriptome assembly of mouse cecum samples

**Fig 4:**
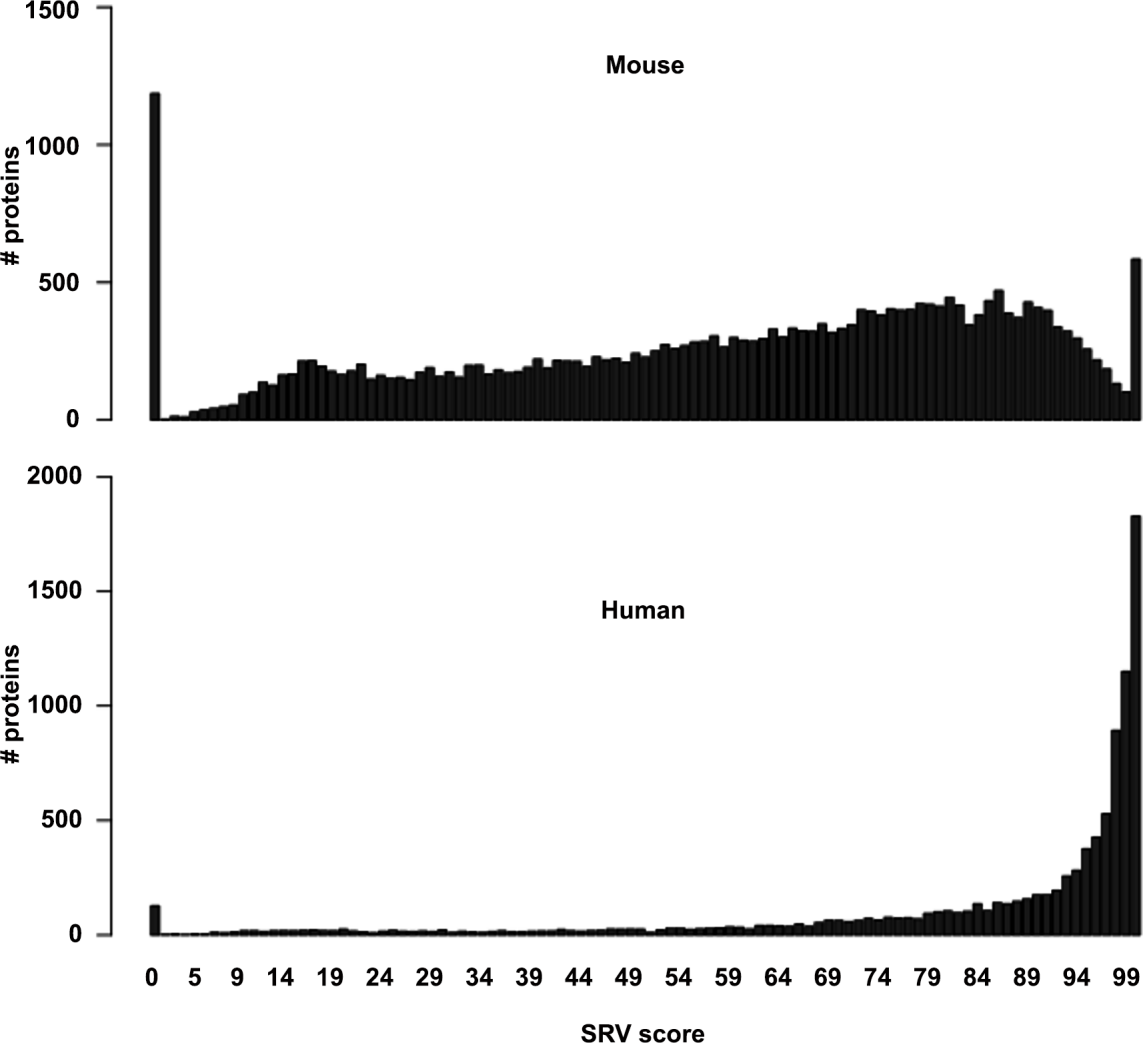
Similarity score distributions of predicted Mouse and Human microbial community proteins to known proteins. Translated proteins were aligned to the NCBI nr protein database and binned according to their SRV score. The SRV score represents the bit-score of the best hit divided by the maximum obtainable bit-score [61].

High protein diets are suggested as effective weight loss regimes and therefore would fit in a successful strategy to achieve a long-term weight loss for a positive effect on health and to decrease obesity and associated metabolic disorders [35]. To assess whether the here developed metatranscriptome pipeline can be used to study the effects of such diets on a GI tract community, four C57BL/6J mice were fed a high-fat high-protein diet for 12 weeks [27]. At the end of the intervention, cecal content was obtained, and the microbial activity present in these samples was analysed by RNA-Seq. Initially, analyses of the mRNA reads employed a previously developed direct alignment approach [10]. However, due to the large evolutionary distance between mouse and human microbiome sequences, and the more limited availability of mouse microbiota associated reference genomes, this procedure resulted in functional and taxonomic information of low resolution (S3 File). To increase the functional and taxonomic resolution the here developed assembly workflow was applied. To monitor precision and recall in this complex and mostly unfamiliar microbial dataset, an RNA-Seq dataset from a well-characterized human small intestinal community [10] was used as a spike-in control and co-assembled with the mouse RNA-Seq data. This led to a *de novo* assembly of 24077 contigs and allowed for the prediction of 36012 partial and full length ORFs within these contigs. Of these ORFs, 25897 were exclusively derived from the mouse datasets, whereas 9707 were assembled exclusively from the human dataset. A total of 407 hybrid ORFs were assembled consisting of reads obtained from both data sources. Virtually identical results were obtained for the mouse-derived RNA-Seq data when the mouse reads were assembled separately (results not shown). This suggests that a substantial increase in microbial complexity essentially did not influence the outcome of the mouse assembly. Of the RNA-Seq mRNA reads derived from the human small intestine sample, 85% was captured in an assembly whereas this fraction was 61–71% for the mRNA reads derived from the four individual mouse cecum samples. The fraction of the reads that was assigned to an ORF was 80% and 45–55% for the human small intestine sample and mouse cecum samples, respectively (Fig 4 and S3 File).

**Fig 5:**
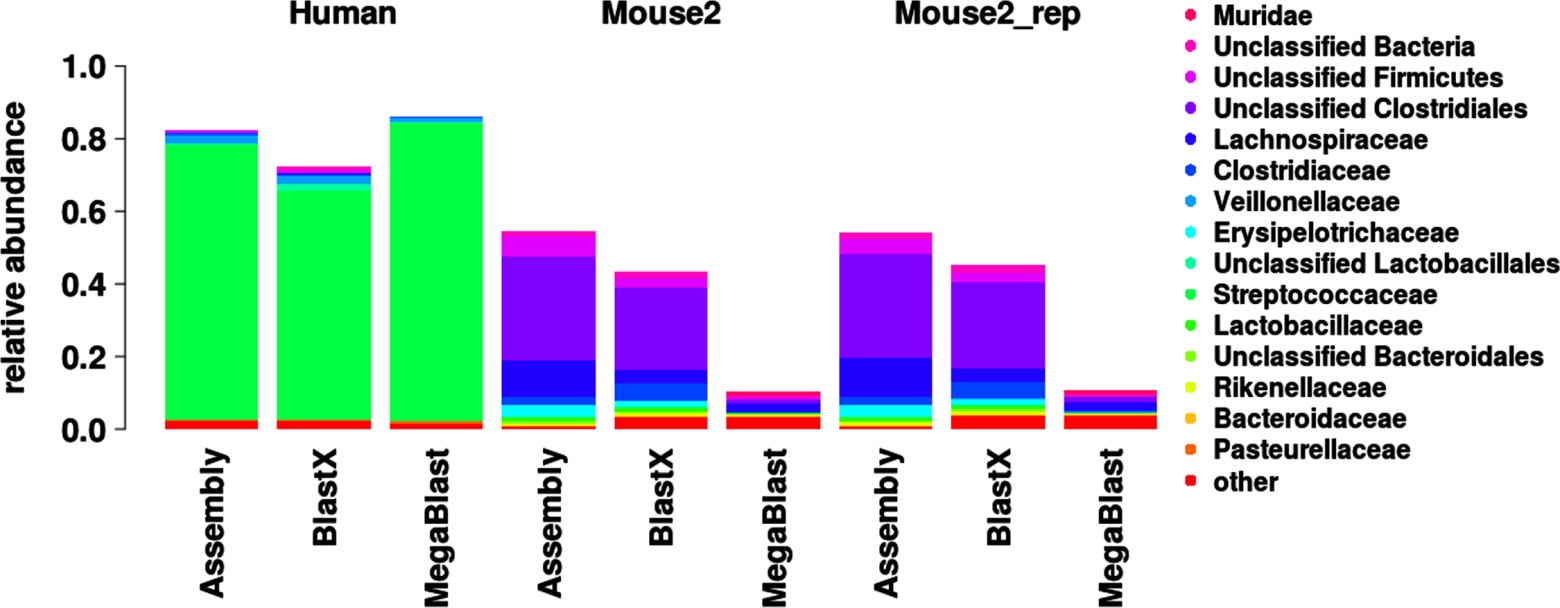
Taxonomic composition of the transcriptome using three different methods. Reads for three samples were assigned to family level using *de novo* assembly, blastx and megablast.

For taxonomic classification the here developed two-step procedure was applied. From the full set ESCG derived marker proteins were identified and used to limit the taxonomic search spaces. Alignment of the full set translated proteins against a thus restricted NR database showed a high level of sequence identity between translated proteins derived from the spike-in control sample and human GI bacterial proteins in the NR database and a low level of sequence identity between mouse GI bacterial proteins and NR bacterial proteins (Fig 5). Further taxonomic classification of proteins from the two environments showed that 93% of the human small intestine protein sequences could be classified at family level, while only 48% of the mouse cecum bacterial protein sequences could be classified at this rank. The high level of sequence identity between proteins derived from the *de novo* assembly of the spike-in control and human GI bacterial proteins in the NR database therefore suggested that overall the assembly was accurate.

### Validation of assemblies by PCR and Sanger sequencing

To further validate the correctness of the mouse metatranscriptome assembly Sanger sequencig of genomic PCR sequences was used. Twelve transcript assemblies of at least 800 nucleotides were randomly selected for having high and low read coverage of genes expressed in the sample of mouse 2. For each of these assemblies’ two sets of specific primer pairs were designed, and in each case two partially overlapping fragments of the correct size could be amplified using cecal microbial DNA of mouse 2 as template (S2 File). For ten assemblies, Sanger sequencing of the amplified DNA returned nucleotide sequences that aligned with 98%-99% identity with the corresponding assembly and thus confirmed the presence of corresponding DNA sequences in the bacterial metagenome. Two PCR products were shown to be a mixture of amplicons originating from isogenic genes. Eight out of the ten correctly sequenced fragments spanned intergenic regions between two coding regions, thus confirming the correct assembly of the genetic context of these genes.

### Technical reproducibility

Technical replicates of higher sequence depth were available for one mouse sample and for the human small intestine sample, and these were employed to establish the technical reproducibility of the procedure and to assess the level of noisiness in the data analysis pipeline. Results showed that for both the mouse and the human small intestinal sample, essentially the same results were obtained for transcripts of high abundance even though there was a 20-fold difference in sequence depth between the two replicates. With decreasing transcript coverage an increase in noise was observed (S4 File).

### Functional analysis of the mouse cecal community

**Fig 6:**
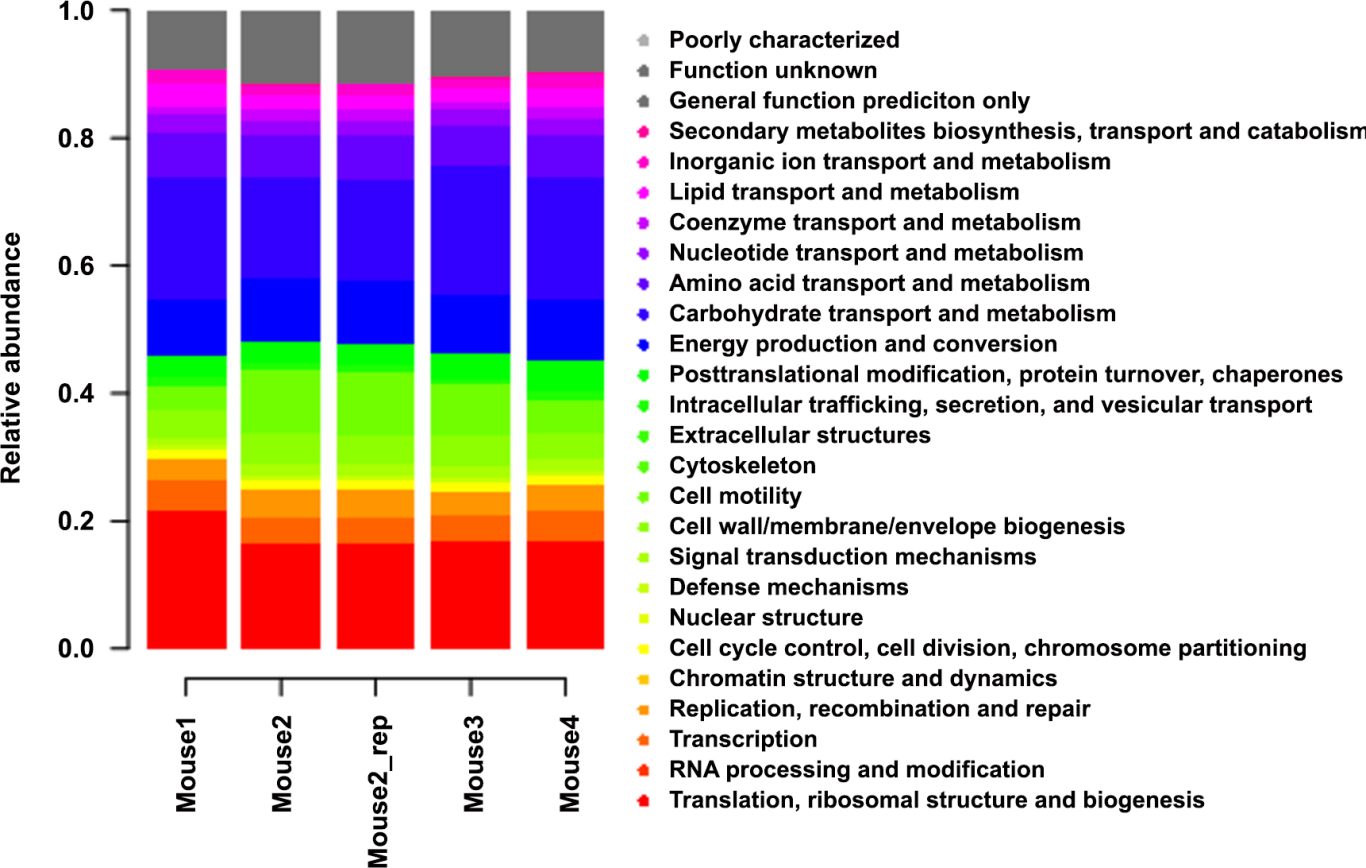
Distribution of COG functional categories of the mouse cecal metatranscriptome.

Using the pipeline, metatranscriptome profiles of cecal microbial communities were obtained from four C57BL/6J mice fed on a high-fat high protein diet [27]. For those four individual communities, the relative expression of COG functional categories was compared. Proteins were labelled according to the COG ontology system [36] and expression levels of proteins belonging to the same category were extracted and lumped (Fig 6). Although the taxonomic profiles of the cecal samples of the four individual mice showed clear differences in relative abundance and distribution of phylotypes the overall COG activity profiles of the four communities were highly similar (0.984–0.999 Pearson correlation, Fig 6).

**Fig 7:**
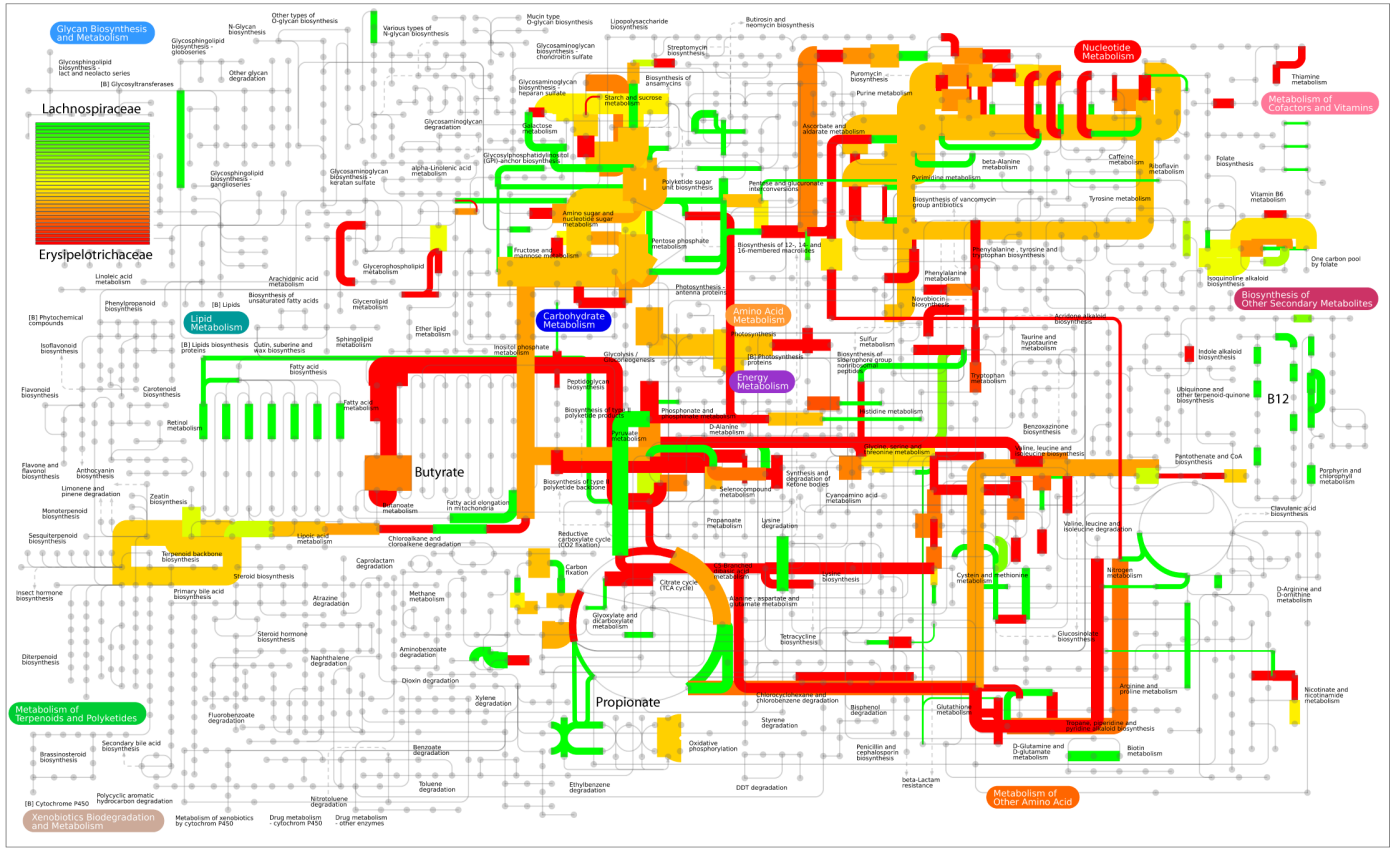
Metabolic pathways mapping of *Lachnospiraceae* and *Erysipelotrichaceae* expression profiles. Relative contribution of each family (green *Lachnospiraceae*, red *Erysipelotrichaceae*) are color scaled. Line-width indicates the total amount of reads mapped to the corresponding KEGG ortholog (log scaled).

To determine whether bacteria from different families fulfilled different roles, homologous proteins of the samples from the C57BL/6J mouse cecum belonging to the four bacterial families with highest number of assigned transcripts, namely the *Clostridiaceae*, *Lachnospiraceae*, *Erysipelotrichaceae* and *Lactobacillaceae*, were annotated using KO identifiers [37], and their cumulative family-specific expression levels were mapped using ipath [38] (S5 File). For the four families the results showed distinct differences in their metabolic profile. For example *Lachnospiraceae* seemed to be active in propionate formation and vitamin B12 biosynthesis, while *Erysipelotrichaceae* appeared to be active in butyrate formation (Fig 7). The *Lactobacillaceae* metabolic activity was found to be mainly oriented towards the production of acetate and lactate, a well-established metabolic feature of this bacterial family. Finally, members of the *Clostridiaceae* family did not display a very clear metabolic activity pattern but compared to the other three families appeared to consistently express amino acid degradation pathways at a higher level. The results show that although no close by reference genomes are available for these organisms, robust functional profiles of these community members can still be obtained. Further studies should give insight into dietary effects on community composition and microbial metabolic activity.

## Conclusions

We have designed and validated a *de novo* metatranscriptome assembly pipeline by using the existing assembler IDBA-UD and performing PCR and sequencing validations on a sample. The pipeline is suitable for analysis of transcriptome data from microbial ecosystems that harbor a degree of diversity such as those encountered in the GI-tract. With this *de novo* metatranscriptome assembly pipeline mRNA reads obtained from RNA-Seq can be assigned to a protein function and a taxonomic rank with high precision. Taxonomic classification on lower ranks remains a major issue for unfamiliar ecosystems harboring many novel species at variable evolutionary distances.

When dealing with familiar ecosystems composed of species that are well represented by complete catalogs of reference sequences at a small evolutionary distance, an assembly strategy will be less efficient due to the required minimal transcript coverage for assembly. However in a community with species that are not well represented in the genome databases, the *de novo* assembly pipeline can outperform direct read alignment methods due to a significant increase in information content of the assembled contigs, which can bridge the phylogenetic distance.

## Materials and Methods

### Ethics statement

The animals used in this study are previously described by Schwarz and co-authors [27]. All animal experiments were approved by the Animal Experimentation Board at Wageningen University (record #2010017) and carried out according to the guidelines of the European Convention of Vertebrate Animals Used for Experimentation, under European Council Directive 86/609/EEC dated November, 1986.

### RNA extraction, mRNA enrichment, cDNA synthesis and Illumina sequencing

The cecal intestinal content was collected from four mice on a high fat high protein diet at 10 weeks during the dietary intervention study previously described by Schwarz and co-authors [27], snap frozen in liquid nitrogen and stored at -80°C. RNA was extracted from 0.1–0.2 grams of cecal content. The content was re-suspended in 500 μL ice-cold TE buffer (Tris-HCL pH 7.6, EDTA pH 8.0). Total RNA was obtained via the Macaloid-based RNA isolation protocol [10,39] with in addition the use of Phase Lock Gel heavy tubes (5 Prime GmbH, Germany) during the phase separation. The RNA purification was done with the RNAeasy mini kit (Qiagen, USA), including an on-column DNAseI (Roche, Germany) treatment [39]. The total RNA was eluted in 30 μL ice-cold TE buffer, and the RNA quantity and quality were assessed using a NanoDrop ND-1000 spectrophotometer (Nanodrop Technologies, Wilmington, USA) and Experion RNA Stdsens analysis kit (Biorad Laboratories Inc., USA), respectively. MRNA enrichment was performed by using the mRNA enrichment kit MICROBExpress™ (Ambion, Applied Biosystems, Nieuwerkerk a/d Ijssel, The Netherlands) using the manufacturer’s protocol. RNA quantity and quality were assessed as described above to determine the efficiency of the mRNA enrichment. Double stranded cDNA was synthesized from one μg of the enriched mRNA sample with the SuperScript® Double-Stranded cDNA Synthesis kit (Invitrogen, The Netherlands), with addition of SuperScript® III Reverse Transcriptase (Invitrogen, The Netherlands) and random priming using random hexamers (Invitrogen, The Netherlands) [10,40,41]. To remove the RNA an RNAse A (Roche, Germany) treatment was preformed, followed by phenol-chloroform extraction of the cDNA and ethanol precipitation. The product was checked on gel and 3 to 8 μg of cDNA was sent for sequencing (GATC Biotech, Germany). Single read Illumina libraries were prepared from the double-stranded cDNA according to the ChiP protocol with insert sizes between 200–300bp. Sequencing was done with an Illumina Hiseq2000, and each sequence library was barcoded and sequenced at 5pM concentration using the single-end protocol. In total the amount of reads was between 700k and 177 M per sample. The data set supporting the results of this article is available in the NCBI small reads archive (sra) repository, under accession number SRX611064.

### PCR Sequencing of a representative selection of assembled contigs

Amplicons targeting a representative selection of assembled contigs were generated by PCR. Primer sets were designed with the NCBI’s primer blast [42] using an optimal melting temperature between 59–61°C with the potential to amplify fragments of around 800 bp (S2 File). Amplicons were generated in two runs. In the first PCR run amplicons from two primer combinations were generated in multiplex reactions, and checked for expected amplicon size. PCRs were performed in a total volume of 25 μl with the FastStart Taq DNA polymerase (Roche), a denaturation of 95°C for 30 seconds an annealing temperature of 60°C for 40 seconds, and elongation for 30 seconds at 72°C, and run for 30 cycles, where the size of the PCR products was confirmed by gel electrophoresis. In the second PCR only the two outer primers were used resulting in the largest obtainable amplicon per contig. The Phusion Hot Start II High-Fidelity DNA polymerase (Thermo Scientific) was used for amplification in a total volume of 50 μl during 35 cycles, consisting of denaturation at 98°C for 10 s, annealing at 60°C for 20 s, 72°C for 50 s for elongation. The size of the PCR products was confirmed by gel electrophoresis and amplicons were sent for Sanger sequencing from both the forward and reverse primer.

### Data filtering

The data was filtered for ribosomal RNA sequences, adapter sequences and low-quality reads using dedicated tools. SortMeRNA (version 1.2) [43] was used to rapidly filter out rRNA sequences using the precompiled databases for eukaryotes, bacteria and archaea. TruSeq adapter sequences were removed from the reads with cutadapt [44]. Initial results showed a high bias of adenines in the trimmed sequences and therefore all trimmed sequences were discarded. The remaining reads where quality (phred >30) and poly A tail edge trimmed using PRINSEQ (lite-version) [45]. Reads smaller than 50 nucleotides were discarded.

### Assembly, annotation and classification

Assemblies were performed using the assemblers’ default setting [46–52]. When required kmer size was set to 31 ORF calling was performed using prodigal 2.60 with the meta procedure [53]. Functional annotation was performed using Interproscan5 with standard settings for all potential output and the KEGG automated annotation server using the SBH method against the default reference set [37,54]. COG annotation was performed by rpsblast (v2.2015) against the NCBI COG database (2-2-2011) with a minimum E-value of 0.0001. Reads were mapped using bowtie2 [55] and expression levels for each predicted protein were extracted using samtools [56] and BEDtools [57]. The first step in the taxonomic classification of the predicted ORFs was identifying all the essential single copy genes and using these to determine the taxonomic search space. Proteins were aligned against the NR database using blastp followed by MEGAN classification [34,58]. A list of GI protein identifiers belonging to the ESCG identified bacterial orders was retrieved via an E-utilities query [59]. The second step was to classify the remaining proteins by aligning them against a GI restricted database followed by MEGAN classification. SRV scores were calculated by dividing the bitscore of the best alignment by the bitscore of a self-alignment. Tetra nucleotide occurrence regression coefficients of the mock community members were calculated using jspecies [60].

## Supporting Information

S1 File Supplementary tables. Assembler Performance: Testing of various assemblers and output performance; Filtering: Ribosomal RNA, adapter and quality filtering results. Mock_TNF_hybrids: Number of shared assemblies and tetra nucleotide frequency occurrence regression coefficient between mock members. Coverage_Assembly: Distribution of assembled proteins based on transcriptome coverage. Mock_assembly ouput: Precision and recall for functional assignments. NOD_mouse_taxonomy_output: Precision and recall for taxonomy assignments
(XLSX)

S2 File PCR primers and Sanger sequencing output of amplicons. (XLSX)

S3 File Read assignment of human small intestine- and mouse cecum-derived metatranscriptome samples using alignment and assembly procedures. Taxonomy profiles for all mouse and human samples using blastx, megablast and assembly strategies.
(PNG)

S4 File Comparison of technical replicate. (PNG)

S5 File Ipath mapping of the 4 most abundant bacterial families found in the metatranscriptome. (PDF)
