# Supplementary figures and images for: Functional Profiling of Unfamiliar Microbial Communities Using a Validated De Novo Assembly Metatranscriptome Pipeline
Source: PLoS One. 2016 Jan 12;11(1):e0146423. doi: 10.1371/journal.pone.0146423 (PMC4710500; doi:10.1371/journal.pone.0146423)

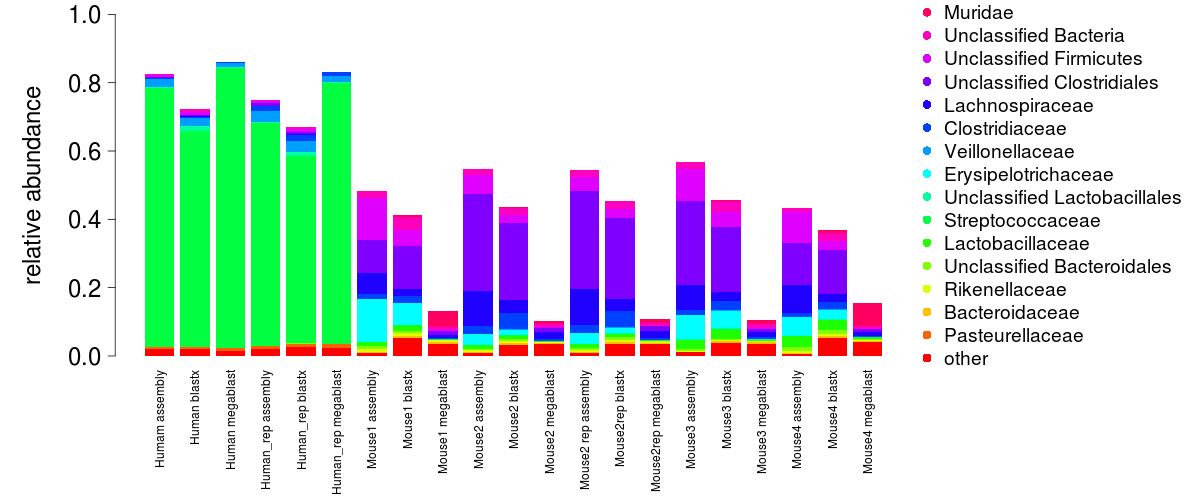

Supplement: S3 File — Taxonomy profiles for all mouse and human samples using blastx, megablast and assembly strategies. (PNG) [file pone.0146423.s003.png]

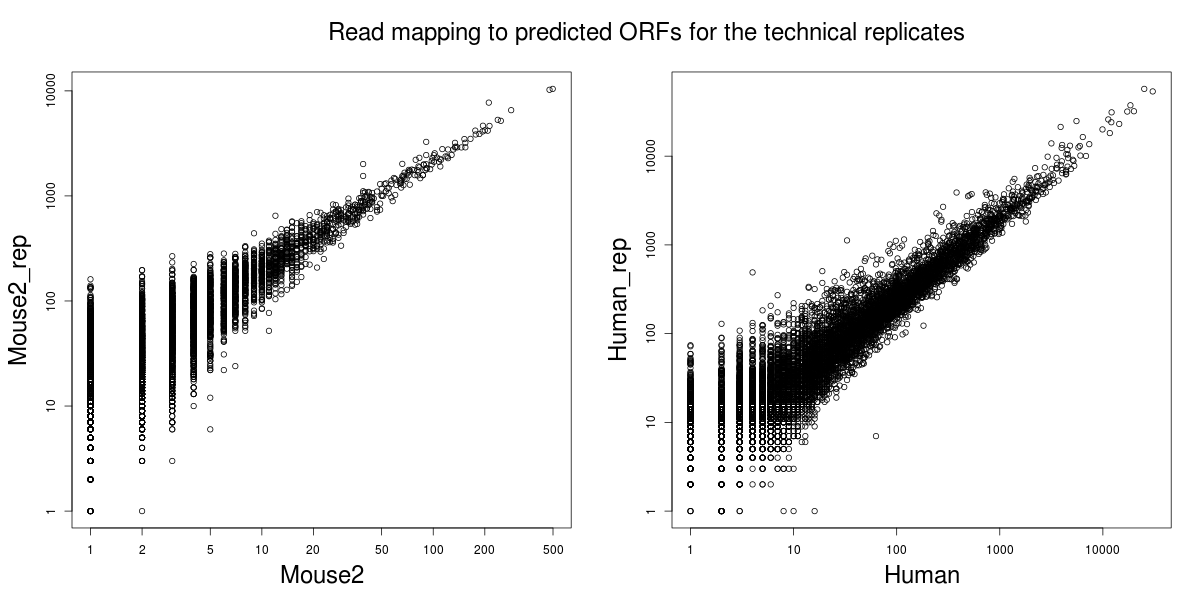

Supplement: S4 File — (PNG) [file pone.0146423.s004.png]

# Clostridiaceae

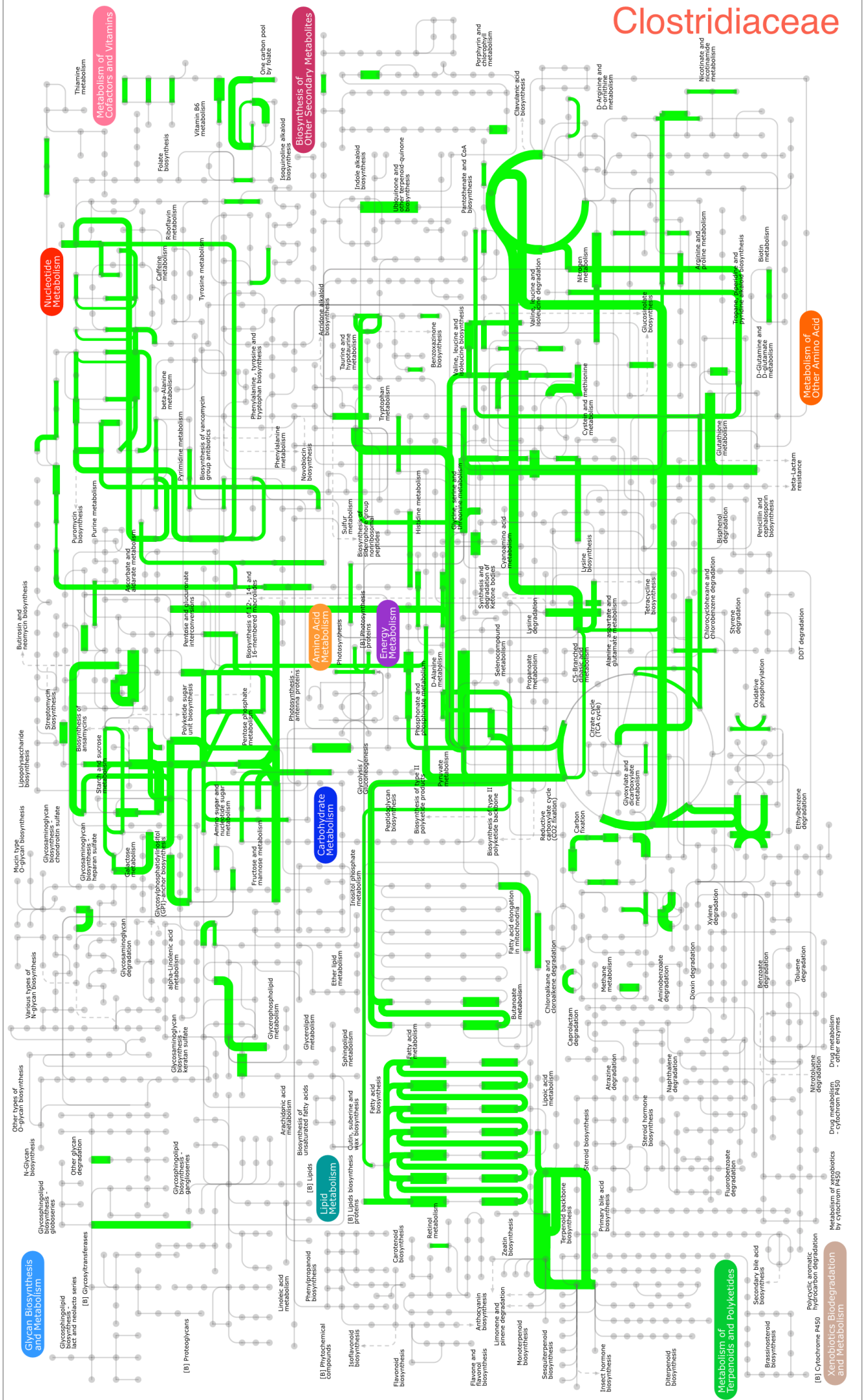

## Lachnospiraceae

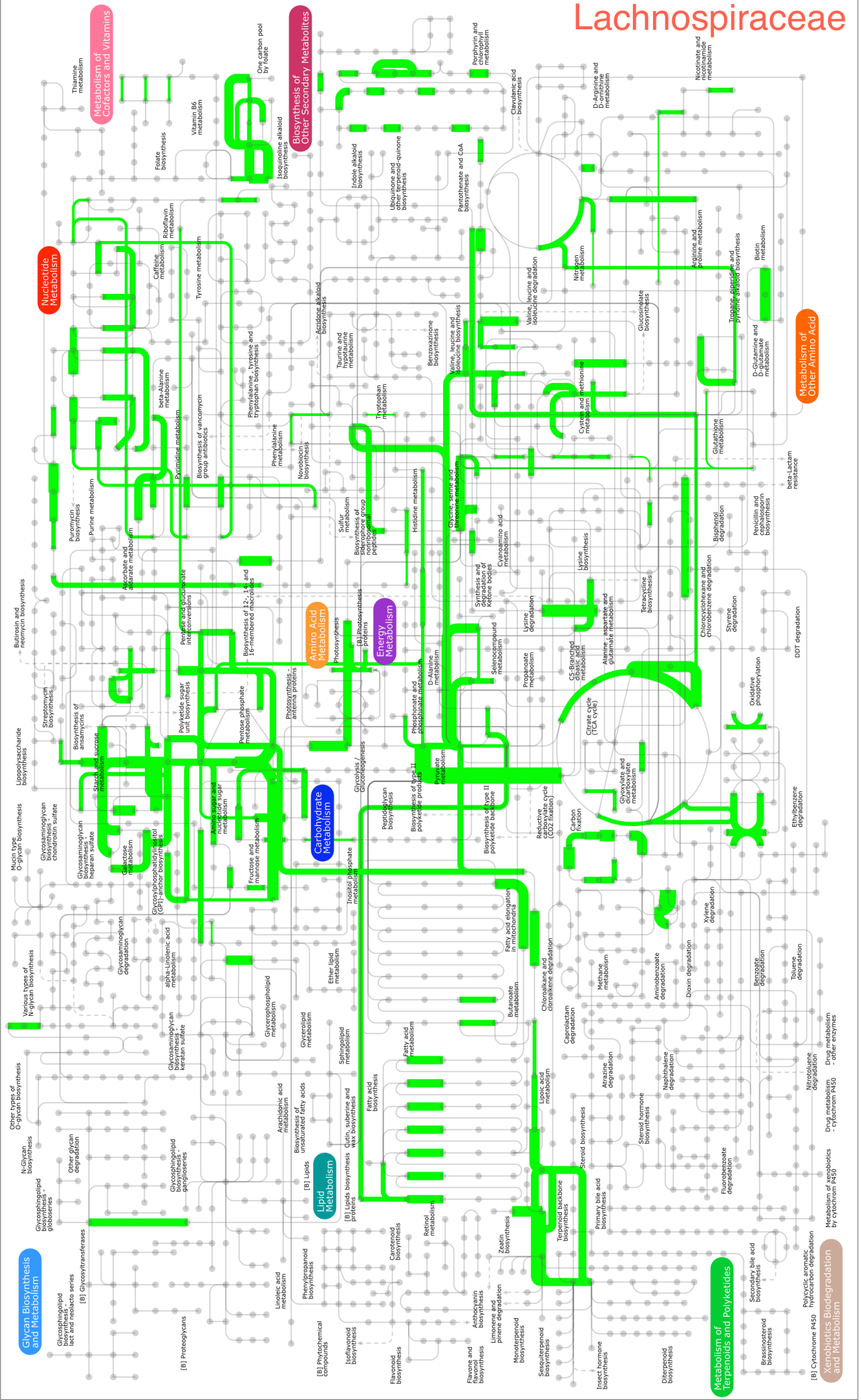

## Erysipelotrichaceae

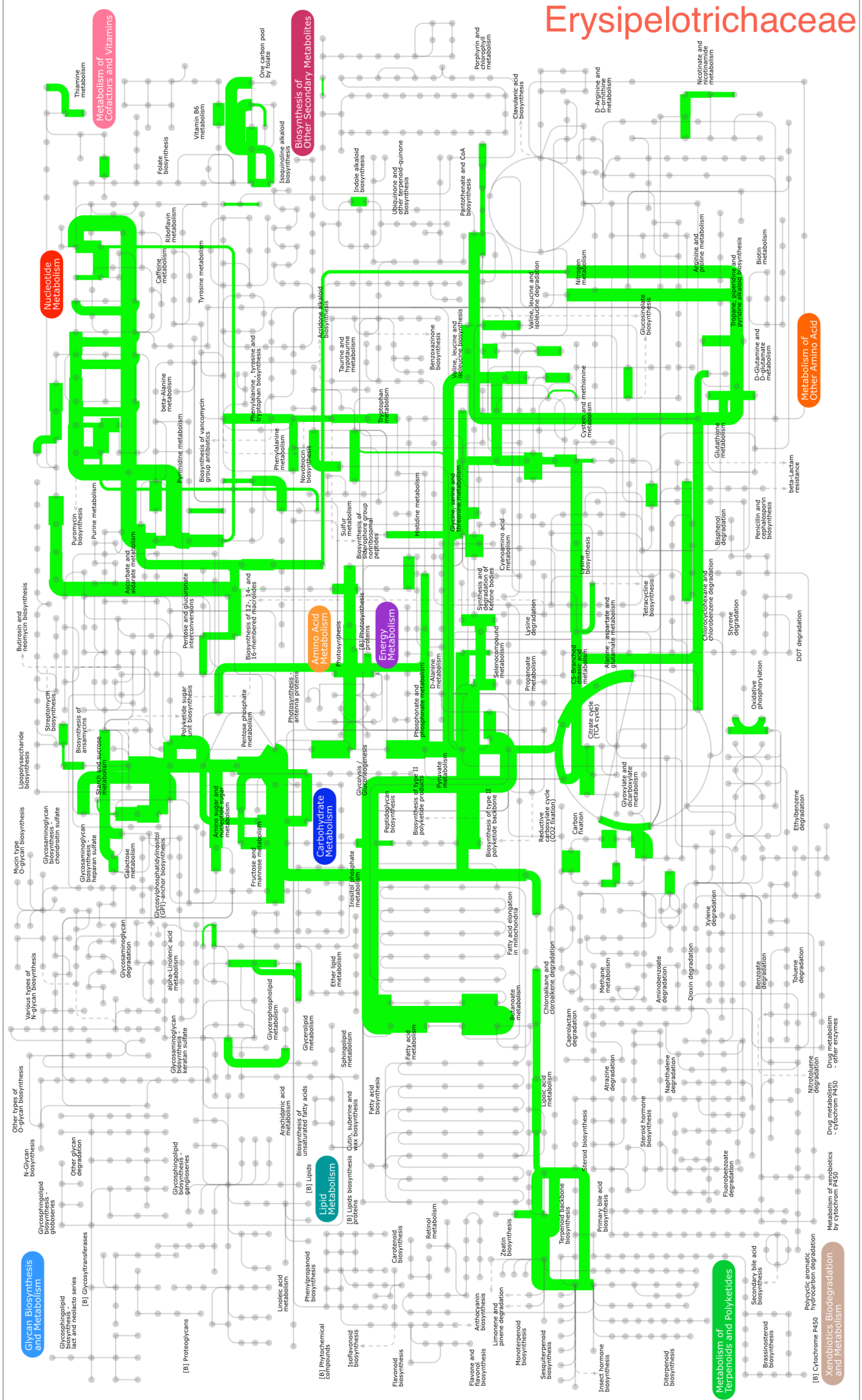

# Lactobacillaceae

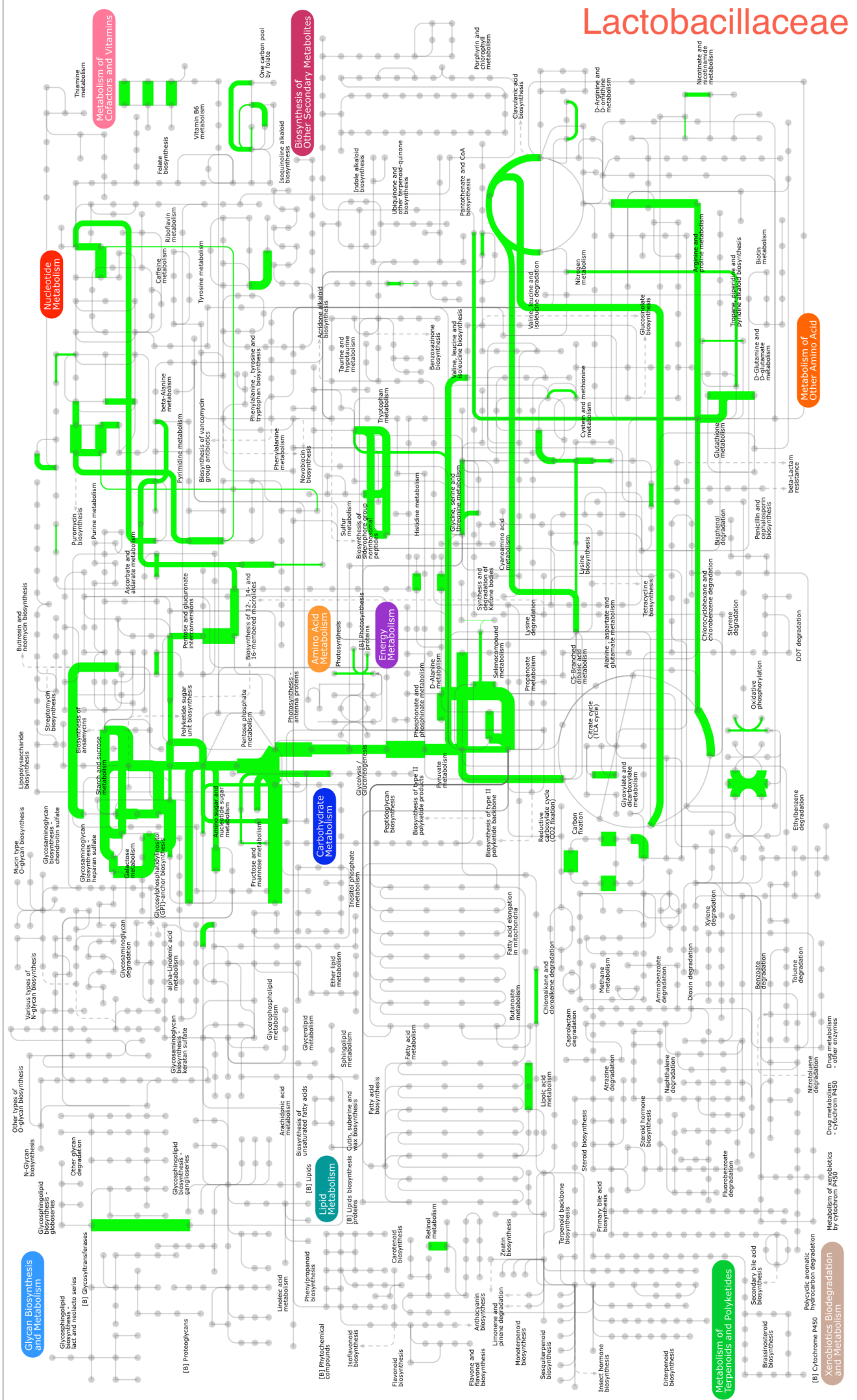

Supplement: S5 File — (PDF) [file pone.0146423.s005.pdf]
